# Population pharmacokinetic analysis of 17-dimethylaminoethylamino-17-demethoxygeldanamycin (17-DMAG) in adult patients with solid tumors

**DOI:** 10.1007/s00280-012-1859-1

**Published:** 2012-03-27

**Authors:** Abdulateef O. Aregbe, Eric A. Sherer, Merrill J. Egorin, Howard I. Scher, David B. Solit, Ramesh K. Ramanathan, Suresh Ramalingam, Chandra P. Belani, Percy S. Ivy, Robert R. Bies

**Affiliations:** 1 Division of Clinical Pharmacology, Department of Medicine, Indiana University School of Medicine, 1001 W. 10th Street, WD W7123, Indianapolis, IN 46202 USA; 2 Roudebush Veteran Affairs Medical Center, Indianapolis, IN USA; 3 University of Pittsburgh Cancer Institute, Pittsburgh, PA USA; 4 Memorial Sloan-Kettering Cancer Center, New York, NY USA; 5 The Translational Genomic Research Institute, Phoenix, AR USA; 6 Winship Cancer Institute, Emory University, Atlanta, GA USA; 7 Penn State Cancer Institute, Hershey, PA USA; 8 National Institute of Health (NIH), Bethesda, MD USA

**Keywords:** 17-dimethylaminoethylamino-17-demethoxygeldanamycin (17-DMAG), 3-compartment model, Heat shock protein-90, Objective function values

## Abstract

**Purpose:**

To identify sources of exposure variability for the tumor growth inhibitor 17-dimethylaminoethylamino-17-demethoxygeldanamycin (17-DMAG) using a population pharmacokinetic analysis.

**Methods:**

A total 67 solid tumor patients at 2 centers were given 1 h infusions of 17-DMAG either as a single dose, daily for 3 days, or daily for 5 days. Blood samples were extensively collected and 17-DMAG plasma concentrations were measured by liquid chromatography/mass spectrometry. Population pharmacokinetic analysis of the 17-DMAG plasma concentration with time was performed using nonlinear mixed effect modeling to evaluate the effects of covariates, inter-individual variability, and between-occasion variability on model parameters using a stepwise forward addition then backward elimination modeling approach. The inter-individual exposure variability and the effects of between-occasion variability on exposure were assessed by simulating the 95 % prediction interval of the AUC per dose, AUC_0–24 h_, using the final model and a model with no between-occasion variability, respectively, subject to the five day 17-DMAG infusion protocol with administrations of the median observed dose.

**Results:**

A 3-compartment model with first order elimination (ADVAN11, TRANS4) and a proportional residual error, exponentiated inter-individual variability and between occasion variability on Q2 and V1 best described the 17-DMAG concentration data. No covariates were statistically significant. The simulated 95% prediction interval of the AUC_0–24 h_ for the median dose of 36 mg/m^2^ was 1,059–9,007 mg/L h and the simulated 95 % prediction interval of the AUC_0–24 h_ considering the impact of between-occasion variability alone was 2,910–4,077 mg/L h.

**Conclusions:**

Population pharmacokinetic analysis of 17-DMAG found no significant covariate effects and considerable inter-individual variability; this implies a wide range of exposures in the population and which may affect treatment outcome. Patients treated with 17-DMAG may require therapeutic drug monitoring which could help achieve more uniform exposure leading to safer and more effective therapy.

**Electronic supplementary material:**

The online version of this article (doi:10.1007/s00280-012-1859-1) contains supplementary material, which is available to authorized users.

## Introduction

The compound 17-dimethylaminoethylamino-17-demethoxygeldanamycin (17-DMAG) is a potential chemotherapeutic treatment for solid tumors due to its ability to degrade oncoproteins by inhibiting heat shock protein-90 [1–5], but its population pharmacokinetic characteristics have yet to be evaluated. An analog of 17-DMAG, 17-(allylamino)-17-demethoxygeldanamycin (17AAG), was the first clinically evaluated heat shock protein 90 inhibitor [6]. However, 17-DMAG has more pharmacologically desirable pharmacokinetic (PK) and pharmacodynamic (PD) profiles because of its reduced metabolic liability, lower plasma protein binding, increased water solubility, higher oral bioavailability, reduced hepatotoxicity and superior antitumor activity compared with 17AAG [5, 7–9]. Preclinical data suggest that, although both 17-DMAG and 17-AAG are excreted primarily through the hepatobiliary system, large differences exist between them in their extent of plasma binding and metabolism [10].

Phase I data indicate that the PK of 17-DMAG is linear, with both the area under the 17-DMAG concentration versus time curve and the maximum concentration increasing proportionally with dose escalation [8]. However, high inter-individual variability in exposure to 17-DMAG despite adjustment of dose to body surface area is a prominent challenge; the coefficient of variation in 17-DMAG exposure can exceed 70 % [11, 12]. A previous 17-DMAG PK model of concentration versus time data [8] employed a noncompartmental method that did not explicitly evaluate within individual variability between occasions and is of limited utility in determining inter-individual variability in drug exposure [13]. The goal of this study was to characterize the population pharmacokinetics of 17-DMAG, and to further explain the nature of the variability in its exposure.

## Patients and methods

### Study setting and participants

The study was conducted at the University of Pittsburgh Cancer Institute (PCI) and Memorial Sloan Kettering Cancer Center (MSKCC). The study protocol was approved by the Institution Review Board of both PCI and MSKCC. All patients gave written, informed consent prior to entering the study.

The study was a phase II assessment of 17-DMAG on patients with a histologically confirmed advanced solid tumor not curable by standard therapies. Patients were excluded if they had abnormal liver function (i.e. liver transaminases ALT or AST higher than 1.5 of the upper limit of normal); abnormal renal function (i.e. blood urea nitrogen and creatinine outside the normal range); or Eastern Cooperative Oncology Group performance status >2. Patients with the following cardiac conditions were excluded because of a prior unconfirmed link of 17-AAG with cardiac toxicity [14]: personal history of long QT syndrome; New York class III or IV heart failure; concurrent use of drugs that prolong QT_C_; personal history of arrhythmia; QT_C_ ≥ 450 ms in males or ≥470 ms in females; poorly controlled angina; uncontrolled dysrhythmias requiring antiarrhythmic drug(s); ejection fraction ≤40 % by multiple gated study; history of serious ventricular arrhythmia (ventricular tachycardia or ventricular fibrillation, three or more consecutive premature ventricular contractions); history of cardiac radiation; or left bundle branch block. Patients with symptomatic pulmonary disease requiring medications or home oxygen were also excluded.

### Drug administration and pharmacokinetic assessment

The Division of Cancer Treatment and Diagnosis (Rockville, MD) supplied 17-DMAG under a cooperative research and development agreement. Sterile, 10 or 50 mg vials of lyophilized 17-DMAG were reconstituted with citrate buffer and mannitol to yield a 5 mg/mL solution. This was further diluted in normal (0.9 %) saline to concentrations between 0.1 and 1 mg/mL and infused intravenously (IV) over 1 h. Antiemetic therapy with oral or intravenous metoclopramide or prochlorperazine was administered prior to drug administration to prevent nausea and vomiting.

Patients at PCI were randomly assigned to one of two schedules, A or B, and doses were adjusted between patients. Schedule A patients received a starting dose of 1.5 mg/m^2^ IV daily for 5 days while patients in schedule B received a starting dose of 2.5 mg/m^2^ IV daily for 3 days. Initial doses were doubled using an accelerated titration schema. In this schema, doses were doubled after one or two patients were accrued per dose level and continued until toxicity higher than grade 2 was observed or a maximum tolerated dose was reached. After the dosage doubling schema was discontinued due to toxicity, the remaining patients were assigned to dose levels that increased in approximately 35 % increments until the maximum tolerated dose was reached. At MSKCC, a range of possible doses was pre-specified and each patient received one single infusion.

PCI patients had serial blood samples collected at baseline, 0.5, 0.92, 1.08, 1.17, 1.25, 1.5, 2.0, 3.0, 5.0, 13.0, 17.0, and 25.0 h after the start of the baseline infusion. Blood samples were also collected prior to the start of the second (25.0 h) and third (49.0 h) infusions. If the patient received 3 doses (schedule B), additional samples were collected at 49.5, 49.92, 50.08, 50.17, 50.25, 51.0, 52.0, and 53.0 h. If the patient received 5 doses (Schedule A), additional samples were collected at the start of the fourth (73.0 h) and fifth (97 h) infusions and at 97.5, 97.92, 98.08, 98.17, 98.25, 98.5, 99.0, 100.0, 102.0 h. MSKCC patients received a single infusion and serial blood samples were collected at 0, 0.5, 0.93, 1.5, 2.0, 4.0, 6.0, 24.0, and 48 h after the start of the infusion. The serial blood samples (5 mL) were collected into heparinized tubes.

Blood samples were centrifuged at 1,000×*g* for 10 min and the supernatant (plasma) was stored at −70 °C. Concentrations of 17-DMAG in the blood were measured using a liquid chromatography/mass spectrometry assay. This assay was developed and validated at the University of Pittsburgh [15].

### Population pharmacokinetic modeling approach

The concentration sampling and dosage history data were combined across the PCI and MSKCC study groups for population PK analysis. Population PK analysis of the 17-DMAG plasma concentration with time was performed using the nonlinear mixed effect modeling software program NONMEM, Version 5.1.1 (GloboMax LLC, Elliot City, Maryland) and the final model was confirmed by re-running the final model in NONMEM, Version 7.1.2

### Base model structure

The initial modeling focused on selecting a base model structure without incorporating any covariates. Linear 2- (ADVAN3, TRANS4) and 3-compartment (ADVAN11, TRANS4) PK models with first order elimination were evaluated. Inter-individual variability (IIV) and between-occasion variability (BOV) random effects were included in the base model and assumed to be log-normally distributed. For example, the value of model parameter *P* after the *k*th dose administered to the *i*th individual, *P*_*i,k*_, is

$$ P_{i,k} = P_{TV} \times e^{{(n_{i}^{(P)} + \kappa_{i,k}^{(P)} )}} $$

where *P*_*TV*_ is the typical value of the model parameter, *η*_*i*_^(*P*)^ is the inter-individual variability, and *κ*_*i,k*_^(*P*)^ is the between-occasion variability.

The BOV was tested on each PK parameter separately by a likelihood ratio test using the objective function values (OFVs) output of NONMEM. The OFV is equal to −2× Log-likelihood (−2LL), and the difference in OFV between models is approximately Chi-square distributed. Each BOV was incorporated one at a time on each parameter in a stepwise forward and then backward elimination fashion. If the OFV did not decrease by at least 3.84 points (*p* < 0.05, 1degree of freedom) after the addition or increase by at least 6.63 points (*p* < 0.01, 1 degree of freedom) after the removal, BOV on that parameter was not considered significant and removed from further consideration in the model. Model structural selection was guided by objective function as well as the Akaike Information Criterion (AIC).

Additive ($y_{{i,j}}  = \hat{y} + \varepsilon _{{i,j}}$), proportional ($y_{{i,j}}  = \hat{y} +  (1 + \varepsilon _{{i,j}} )$), and combined error ($y_{{i,j}}  = \hat{y}_{{i,j}} (1 + \varepsilon _{{i,j}} ) + \varepsilon _{{i,j}}^{\prime}$ ) residual error structures were tested, where *y*_*i,j*_ is the *j*th observed DMAG concentration in the *i*th individual, $\hat{y}_{{i,j}}$ is the corresponding model prediction, and ε_*i,j*_ (and ε_*i,j*_^*′*^) is the residual error which is assumed to be normally distributed with a mean of 0 and a variance of σ^2^ (and σ^*′*2^).

### Covariate assessment

Differences in patients’ demographic characteristics may explain some of the variability in the PK parameter estimates. In this study, the continuous covariates age, albumin, alanine aminotransferase, aspartate transaminase, bilirubin, blood urea nitrogen, body surface area, creatinine, and weight and the discrete covariate sex were tested. The effect of continuous covariates on the typical value of a parameter was modeled using additive, proportional, emax and exponentiated forms. An example of the implementation of a typical continuous covariate (centered, exponentiated) is

$$ P_{TV} = \theta_{1} \exp \left( {\theta_{2} \frac{Cov}{median(Cov)}} \right) $$

where *θ*_1_ and *θ*_2_ are estimated fixed effect parameters and *Cov* is the subject-specific value of the covariate. Categorical (binary) values were assigned to each discrete variable (such as sex), and their effect on PK parameters were modeled as

$$ \begin{gathered} P_{TV} = \theta_{1} \quad {\text{if}}\,Cov = 1 \hfill \\ P_{TV} = \theta_{2} \quad {\text{if}}\,Cov = 2 \hfill \\ \end{gathered} $$

For the final model, each covariate was tested individually using the stepwise forward addition then backward elimination method using the model discrimination criteria previously described for BOV evaluation. In addition, diagnostic plots of observed concentrations versus population predictions, observed concentrations versus individual predictions, weighted residuals versus population predictions, and weighted residuals with time were inspected for systematic deviation.

## Results

### Patient characteristics

A total of 67 subjects (48 PCI and 19 MSKCC) participated in the study (Table 1). The subjects received a median infusion dose of 36 mg/m^2^ (range of 2.2–413 mg/m^2^) and contributed a total of 1,148 17-DMAG plasma concentration measurements (1,000 PCI and 148 MSKCC). No subjects or 17-DMAG concentrations were excluded. The majority of subjects were male (42 vs. 25) and had a median age of 63 years and a median weight of 80.3 kg.

**Table 1:** Patient characteristics

Characteristics	*N* (%)	Median (Range)
Number of patients	67	
Pittsburgh Cancer Institute	48 (72 %)	
Memorial Sloan-Kettering	19 (28 %)	
Number of observations	1,148	21 (7–25)
Pittsburgh	1,000 (87 %)	23 (10–25)
Memorial Sloan-Kettering	148 (13 %)	8 (7–9)
DMAG dose, μg		36,000 (2,200–413,000)
DMAG concentration, μg/mL		203.5 (1–5,542)
Age, years^a^		63 (28–82)
Sex		
Male	42 (63 %)	
Female	25 (37 %)	
Weight, kg^a^		80.3 (48.2–136.5)
Albumin, g/dL^b^		3.8 (2.6–5.1)
Alanine aminotransferase, units/L		22 (10–106)
Aspartate transaminase, units/L		25 (12–75)
Bilirubin, mg/dL		0.5 (0.1–3.0)
Blood urea nitrogen, mg/dL^c^		15 (5–70)
Body surface area, m^2^		1.9 (1.5–2.6)
Creatinine, mg/dL		1.0 (0.6–1.8)

^a^One patient is missing data for age, weight, albumin, alanine aminotransferase, aspartate transaminase, bilirubin, blood urea nitrogen, and creatinine

^b^Albumin measurements were missing for 6 patients

^c^Blood urea nitrogen measurements were missing for 2 patients

### Population pharmacokinetic modeling

A 3-compartment model with first order elimination (ADVAN11, TRANS4) and a proportional residual error best described the 17-DMAG concentration data in this population. Between-occasion variability was significant only on Q2 and V1. None of the covariates showed a significant relationship with the parameter values.

All fixed and random effects were well determined in the final model (Table 2). The population average for the clearance and inter-compartmental clearances were 8.4, 85.1, and 11.6 L/h, respectively. Volumes for the three compartments were 27.4, 66.4 and 142 L, respectively. The inter-individual variability for CL, Q3, V1, V2 and V3 were 53.8, 75.8, 33.0, 50.7, and 67.5 %, respectively. The between-occasion variability on Q2 and V1 were 32.6 and 59.7 %, respectively. A proportional error model was used and the proportional residual error was 16.1 %. Diagnostic plots are available in the supplemental material.

**Table 2:** Parameter values for final model

Parameter	Population estimate (%SE)	Inter-individual variability (%SE)	Between-occasion variability (%SE)
CL	8.4 (11.2)	53.8 % (22.9)	–
Q2	85.1 (9.6)	–	32.6 % (37.2)
Q3	11.6 (13.1)	75.8 % (32.0)	–
V1	27.4 (11.7)	33.0 % (111.9)	59.7 % (31.2)
V2	66.4 (10.1)	50.7 % (23.7)	–
V3	142 (13.5)	67.5 % (37.3)	–
Proportional error	16.1 % (2.7)	–	–

### Effects of inter-occasion variability and between-occasion variability on 17-DMAG exposure

Because of the absence of covariate effects on the model parameters and the wide inter-individual variability in this population PK analysis, the population exposure to 17-DMAG was quantified in a post hoc Monte Carlo simulation. The final model was used to simulate the 95% prediction interval for the area under the 17-DMAG concentration time curve per dose, AUC_0–24 h_, for the five dose infusion protocol with 36 mg/m^2^ doses. The simulated 95 % prediction interval of the AUC_0–24 h_ with both IIV and BOV was 1,059–9,007 mg/L h.

Because of the wide range of exposures due to IIV, a second post hoc analysis was performed to simulate the variability in exposure due to BOV. Typical model parameters (i.e. no IIV) were used to simulate the 95% prediction interval of the AUC_0–14 h_ for the five dose infusion protocol with 36 mg/m^2^ doses. The simulated 95% prediction interval of the AUC_0–24 h_ with BOV only was 2,910–4,077 mg/L h.

## Discussion

In this study, we implemented the first population PK analysis to identify, measure, and characterize the potential sources of variability in 17-DMAG concentrations using data from patients with advanced solid tumors. These analyses suggest that 17-DMAG disposition is best described using a three-compartment, linear model. There were no statistically significant effects of covariates on parameters in the model. The wide inter-individual variability in this model leads to considerable variation in exposure to 17-DMAG between individuals which is consistent with previous studies [11, 12]. But, based on our results, we cannot comment on whether or not this fluctuation contributed to the lack of PK/PD correlation in the previous studies [8, 12]. The challenge of substantial variability in its pharmacokinetic parameters hinders the implementation of 17-DMAG and such wide variations in exposure may reduce efficacy and precipitate toxicity in under-, and over-exposed patients respectively. In addition, the BOV of 32.6 % in inter-compartmental clearance (Q_2_) and 59.7 % variability in the volume of the central compartment did not have a considerable impact on the dose to dose exposure within a given individual. In light of the considerable IIV, this result suggests a role for therapeutic drug monitoring to characterize individual PK characteristics after single dose of 17-DMAG.

The study included only patients with adequate hepatic, hematological, and renal function to ensure safety and adequate drug metabolizing capability. As such, the effects of extreme derangements in hepatic, hematological, and renal function on the pharmacokinetics of 17-DMAG are unknown. In order to effectively capture covariate relationships such as hepatic and renal function on 17-DMAG pharmacokinetics, a wider range of values for the covariates would be necessary. Our strict inclusion/exclusion criteria precluded this. Despite controlling for a relatively homogenous study population, our analysis still contain significant random IIV. Therefore, this model has limited utility for dosage targeting prior to the first dose, as evidenced by the individual predictions (Supplemental Figure 1). However, once a plasma concentration sample is measured, the dosage can be much more precisely tailored at the individual level.

In addition, the model could provide the basis for simulation of future 17-DMAG clinical trials, the selection of optimal sampling points to enhance the capture of inter-individual variability, and the possibility of covariate effects that were undetected in these analyses.

Strengths of this study include multiple dosing, frequent and multiple sampling, appropriate target population (cancer patients), and data from multiple centers to strengthen the conclusions of these analyses.

## Electronic supplementary material

Below is the link to the electronic supplementary material.

Supplementary material 1 (DOCX 95 kb)

Supplementary material 2 (DOCX 15 kb)

Supplementary material 3 (DOCX 11 kb)
