# Supplementary figures and images for: Population pharmacokinetic analysis of 17-dimethylaminoethylamino-17-demethoxygeldanamycin (17-DMAG) in adult patients with solid tumors
Source: Cancer Chemother Pharmacol. 2012 Mar 27;70(1):201–5. doi: 10.1007/s00280-012-1859-1 (PMC3383947; doi:10.1007/s00280-012-1859-1)

**SUPPLEMENTAL MATERIALS**

**Figure 1**


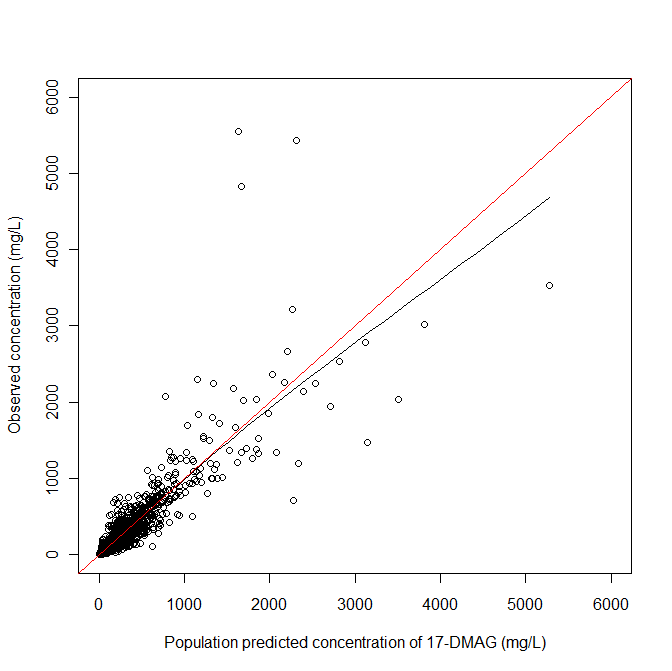

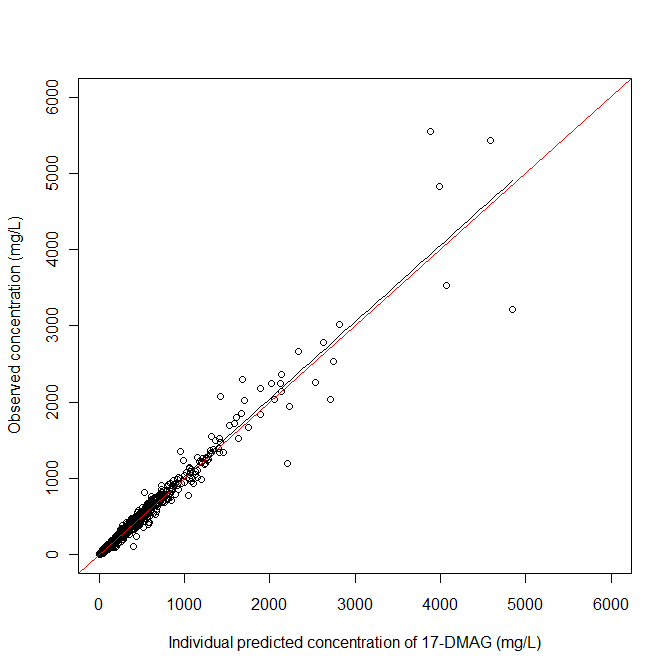


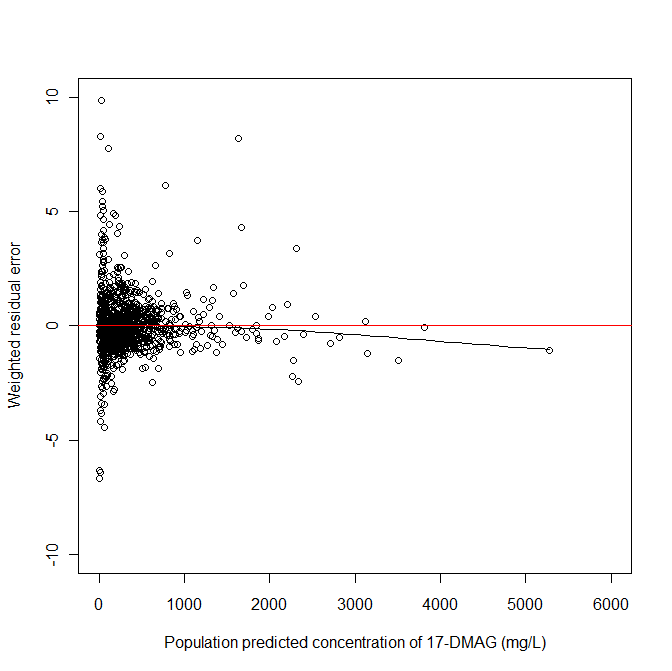

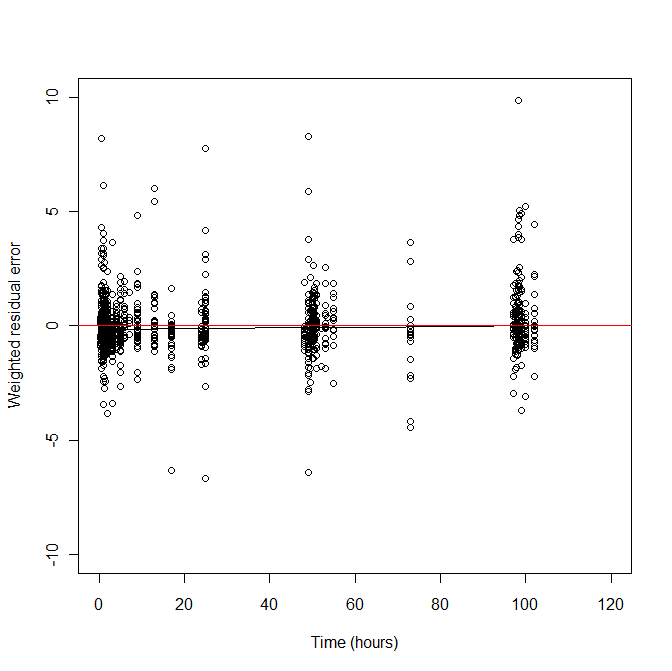

Supplement: Supplementary file 1 — Supplementary material 1 (DOCX 95 kb) [file 280_2012_1859_MOESM1_ESM.docx]
