# Supplementary material for: Population pharmacokinetic analysis of 17-dimethylaminoethylamino-17-demethoxygeldanamycin (17-DMAG) in adult patients with solid tumors
Source: Cancer Chemother Pharmacol. 2012 Mar 27;70(1):201–5. doi: 10.1007/s00280-012-1859-1 (PMC3383947; doi:10.1007/s00280-012-1859-1)
Supplement: Supplementary file 2 — Supplementary material 2 (DOCX 15 kb) [file 280_2012_1859_MOESM2_ESM.docx]

**Figure 2**

(A)

(B)
