# Supplementary material for: Population pharmacokinetic analysis of 17-dimethylaminoethylamino-17-demethoxygeldanamycin (17-DMAG) in adult patients with solid tumors
Source: Cancer Chemother Pharmacol. 2012 Mar 27;70(1):201–5. doi: 10.1007/s00280-012-1859-1 (PMC3383947; doi:10.1007/s00280-012-1859-1)
Supplement: Supplementary file 3 — Supplementary material 3 (DOCX 11 kb) [file 280_2012_1859_MOESM3_ESM.docx]

**FIGURE LEGENDS**

**SUPPLEMENTAL MATERIALS**

**Figure 1**

Diagnostic plots for the final pharmacokinetic model of 17-DMAG. (A) The observed versus population predicted 17-DMAG concentrations; (B) the observed versus individual predicted 17-DMAG concentrations; (C) weighted residual error versus population predicted 17-DMAG concentration; and (D) weighted residual error with time. The black circles are individual data points, the solid black lines are smoothed curves through the data, and the solid red lines are lines are unity lines.

**Figure 2**

17-DMAG plasma concentration vs. time profile for a typical patient who received a median dose of: (A) a 3-dose one-hour infusion protocol of 33,000 μg/dose at Pittsburgh or (B) a single-dose one-hour infusion of 170,000 μg at Memorial Sloan-Kettering.
